# Measuring the prevalence of regional mutation rates: an analysis of silent substitutions in mammals, fungi, and insects

**DOI:** 10.1186/1471-2148-8-186

**Published:** 2008-06-27

**Authors:** Aleah K Fox, Brian B Tuch, Jeffrey H Chuang

**Affiliations:** 1 Department of Biology, Boston College, Chestnut Hill, MA 02467, USA; 2 Department of Biochemistry & Biophysics and Department of Microbiology & Immunology, University of California San Francisco, San Francisco, CA 94143-2200, USA

## Abstract

**Background:**

The patterns of mutation vary both within and across genomes. It has been shown for a few mammals that mutation rates vary within the genome, while for unknown reasons, the *sensu stricto *yeasts have uniform rates instead. The generality of these observations has been unknown. Here we examine silent site substitutions in a more expansive set (20 mammals, 27 fungi, 4 insects) to determine why some genomes demonstrate this mosaic distribution and why others are uniform.

**Results:**

We applied several intragene and intergene correlation tests to measure regional substitution patterns. Assuming that silent sites are a reasonable approximation to neutrally mutating sequence, our results show that all multicellular eukaryotes exhibit mutational heterogeneity. In striking contrast, all fungi are mutationally uniform – with the exception of three *Candida *species: *C. albicans, C. dubliniensis*, and *C. tropicalis*. We speculate that aspects of replication timing may be responsible for distinguishing these species. Our analysis also reveals classes of genes whose silent sites behave anomalously with respect to the mutational background in many species, indicating prevalent selective pressures. Genes associated with nucleotide binding or gene regulation have consistently low silent substitution rates in every mammalian species, as well as multiple fungi. On the other hand, receptor genes repeatedly exhibit high silent substitution rates, suggesting they have been influenced by diversifying selection.

**Conclusion:**

Our findings provide a framework for understanding the regional mutational properties of eukaryotes, revealing a sharp difference between fungi and multicellular species. They also elucidate common selective pressures acting on eukaryotic silent sites, with frequent evidence for both purifying and diversifying selection.

## Background

Though neutral mutation rates were once considered to be uniform, it has been discovered that they can vary not only from one species to the next but also within a single genome [1]. Within-genome heterogeneity has been demonstrated in a few mammalian species, namely human, chimp, mouse, rat, dog, and cow [2-8]. In contrast, uniform mutation rates have been observed only in the phylogeny of the *sensu stricto *yeasts *S. cerevisiae, S. paradoxus, S. bayanus*, and *S. mikatae *[9]. The reason for this differing mutational behavior is not understood, and little is known about regional biases in other species. Characterization of regional biases in a broader range of species would improve our understanding of genomic mutational properties. It would also aid in the calibration of phylogenetic footprinting methods, which are important for identifying sequences under purifying selection [10].

Most previous studies of mutation rate variation have focused on mammals. Regional effects in the rodent-human clade have been detected, for example, by analyzing the correlations of distinct pseudo-neutral quantities such as SNP density, indel density, substitution rate in ancestral repeats, and substitution rates in silent sites [11]. Regional effects have been further characterized via the length scales at which nearby sequences are correlated, as well as via correlations within single genes [2]. Proposed explanations for heterogeneous mutation rates have included variations in base composition, recombination, gene density, and pattern of gene expression, though the causal relationships remain uncertain [1].

An important step toward understanding the causes of mutational heterogeneity would be to characterize regional mutation rates in a more diverse sampling of species. The diversity of available genomes could aid in distinguishing different types of effects. For example, the length scale of mammalian regional variation (~10 Mb)[2] is larger than a typical yeast chromosome (1 Mb). Are long chromosomes a requirement of the mechanism underlying regional mutation heterogeneity? A single example of regional heterogeneity in a yeast species would suggest this is not the case.

In this work, we use synonymous sites to analyze regional mutational biases in 20 mammalian, 27 fungal, and 4 insect genomes. Such sites can be altered without affecting the encoded amino acid sequence and are thus typically less likely to be under selective pressure. This property has made them a common dataset for measuring neutral mutation rates[12]. Although it has been shown that some synonymous sites have functional roles (reviewed in [13]), only a minor fraction are likely to be under selection pressure (also discussed in [13]). For example, among the *sensu stricto *yeasts, only 8% of genes (mostly under codon usage selection) exhibit silent substitution rates that deviate from the uniform neutral mutational background[9], and analysis of these genes shows that they perturb the overall genome-wide silent substitution rate by only 3%.

Using silent sites as a proxy for neutral sequence, we answer the following questions: Do all mammals exhibit heterogeneity in their mutation rates? And, do all fungi exhibit uniformity? Studying these phylogenies together provides a valuable contrast. For instance, we unexpectedly find that while most fungi have uniform mutation rates, *C. albicans *and its closest relatives do exhibit regional heterogeneity. In contrast, we find that all mammals and several insects have heterogeneous mutation rates. Because our approach involves distinguishing the neutral pressures that influence silent sites, our study also naturally reveals genes whose silent sites behave unusually with respect to the mutational background in each species. Interestingly, we find two strong themes governing such genes. First, we find evidence for purifying selection on synonymous sites in genes associated with RNA binding or DNA binding, in both mammals and fungi. Second, we find evidence that diversifying selection has regularly influenced the silent sites of receptor genes, particularly in the mammals.

## Results

There are many types of sequences in the genome which may be used to determine the neutral mutation rate. In this work we focus on synonymous sites, in particular fourfold degenerate sites, from coding regions in 51 species. To isolate lineage-specific effects, pairs of species that are closely related were chosen based on recently published yeast [14] and mammalian phylogenies [15,16]. Each gene from one species was mapped to an ortholog in the second species and the two corresponding sequences were aligned. Synonymous substitution rates were calculated for each orthologous gene pair by counting the fraction of observed substitutions at fourfold sites and then normalizing to a z-score value (referred to as *r*) under the assumption that all synonymous sites mutate independently at the genome-average rate (see Methods). Under this assumption, a genome with a uniform mutation rate will exhibit unit standard deviation in its *r *distribution, while a heterogeneously mutating genome will exhibit a larger standard deviation [2].

### Most fungi have uniform mutation rates

**Figure 1:**
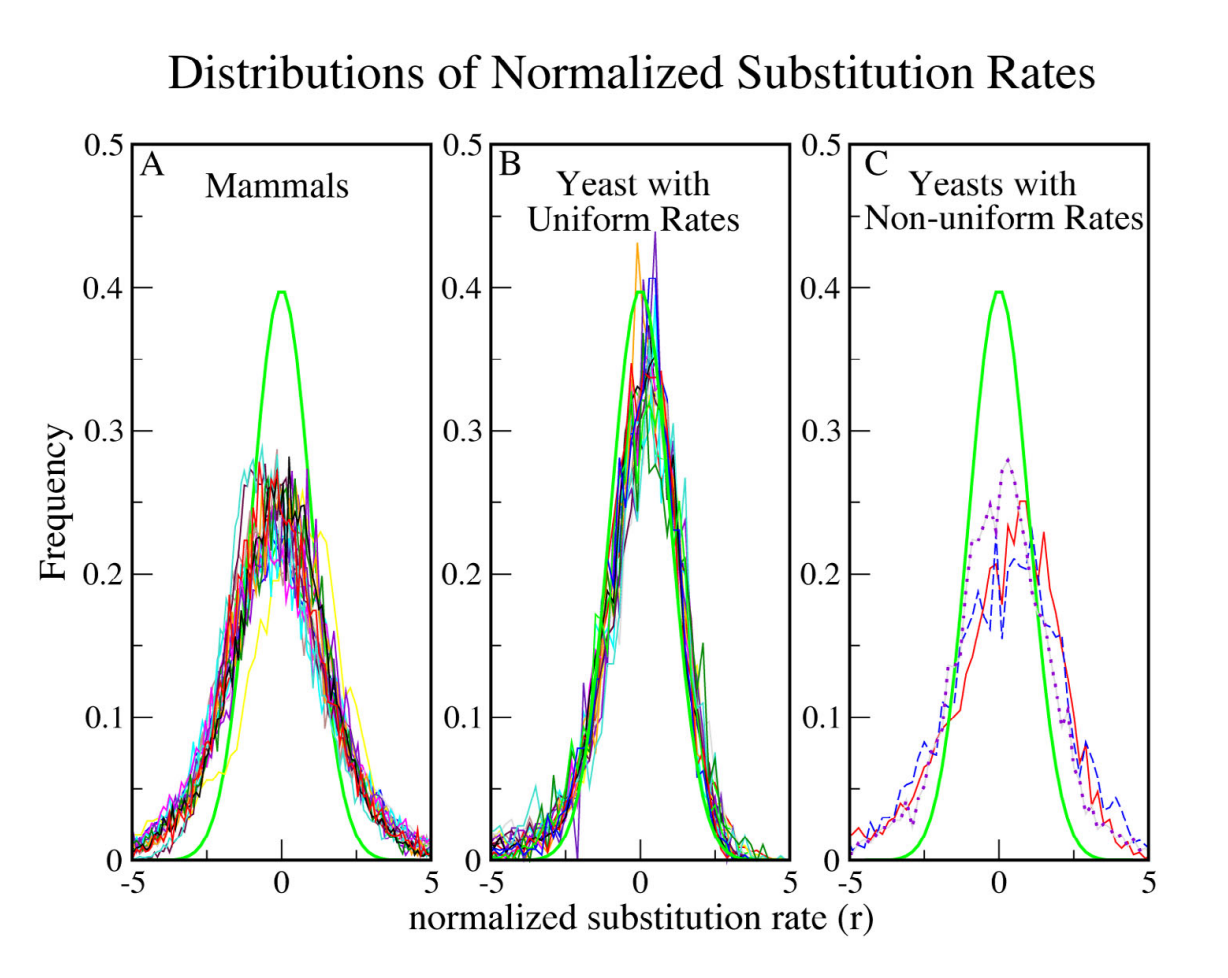
**Distributions of normalized 4-fold substitution rates**. These graphs indicate that most mammals have heterogeneous mutation rates and most fungi have uniform mutation rates. The normal Gaussian distribution (thick green line) is what would be expected if all 4-fold sites in each gene have an equal and independent probability of being substituted. (a) All mammalian distributions have a bias toward high and low substitution rates, consistent with regional mutation biases in mammalian genomes (b) Most fungal distributions fit more closely to the Gaussian, except for a tail of genes with low substitution rates stemming from codon usage selection. (c) The distributions of *C. albicans – C. dubliniensis *(dotted purple line)*, C. tropicalis – C. dubliniensis *(solid red line), and *N. crassa – C. globosum *(dashed blue line). These species pairs each have a wider distribution, similar to the mammalian distributions.

The distribution of normalized substitution rates (*r*) provides a test of whether substitution rates are uniform throughout a genome. In genomes with uniform rates such as *S. cerevisiae*, the *r *distribution is very close to the theoretical prediction of a Normal distribution with unit standard deviation (σ = 1) [9]. However, in mammalian genomes, the width of the *r *distribution is considerably larger, due to the tendency of sites in the same gene to be subject to similar regional mutation rates (Figure 1a).

**Figure 2:**
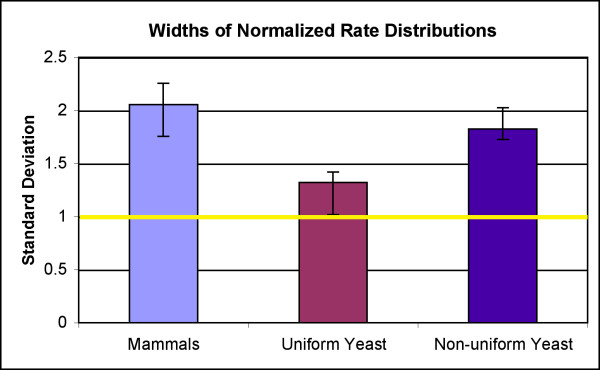
**Comparison of the widths of the rate distributions for fungi and mammals**. The yellow line at σ = 1 shows the expected standard deviation for a Normal distribution. The mammals have an average σ of 2.056, indicating their bias toward both high and low rates – consistent with regional mutation rates. There are three fungal pairwise comparisons (*C. dubliniensis/C. albicans, C. tropicalis/C. dubliniensis*, and *N. crassa/C. globosum*) which also have wide rate distributions, with an average σ of 1.825 (see Figure 1c). The species pairs from the remaining 22 fungi have a noticeably lower value of σ (average σ = 1.32, range = [1.02 – 1.40]), consistent with their having uniformly mutating genomes. Plotted values indicate the average σ in each group and horizontal lines mark the range.

In the current fungal dataset (27 species, 26 closely related species pairs; a phylogeny is available in Additional file 1), we found that the *r *distributions for 22 of the 27 species (23 species pairs) are a good fit to the Normal distribution (Average σ = 1.32, range = [1.02 – 1.40], Figure 2). This indicates that there are generally not regions of high or low neutral mutation rate within these genomes (Figure 1b). These 22 fungal species (All species other than *C. albicans, C. dubliniensis, C. tropicalis, N. crassa*, and *C. globosum*) do have a slight excess of genes at negative *r*. That group is largely comprised of ribosomal and metabolism genes, consistent with genes known to be under selection for codon usage bias [9]. Of the 432 genes with *r *≤ -2 in the *S. cerevisiae-S. bayanus *comparison, 201 (46.5%) have a ribosomal or metabolism GO annotation. Such codon usage selection is clear across the fungi. For example, in the distinct lineage *D. hansenii-C. guilliermondii*, 138 of 253 genes (54.5%) with *r *≤ -2 also map to ribosomal or metabolism GO categories.

The inference that neutral mutation rates do not vary across these 22 fungal genomes is further supported by a neighboring gene analysis. For each pair of species, we calculated the Pearson correlation of *r *values for neighboring genes (see Methods). These results are shown in Table 1. We found no apparent correlation for any of the species pairs chosen from among these 22 genomes. The same results were obtained irrespective of which species in the pair was used to specify gene locations.

**Table 1:** Pearson correlation of substitution rates between neighboring fungal genes, for 26 species pairs among 27 fungi.

Pair #	Species 1	Species 2	Avg. divergence	# orthologs	Pearson Correlation	p-value
*1	*S_cerevisiae*	*S_paradoxus*	*0.2592*	*4434*	*0.0234*	*0.1188*
*2	*S_cerevisiae*	*S_bayanus*	*0.4709*	*3781*	*0.0305*	*0.0606*
3	S_castellii	S_mikatae	0.6585	3247	0.0029	0.8649
4	K_lactis	C_glabrata	0.6545	4077	-0.0135	0.3887
5	D_hansenii	C_lusitaniae	0.6926	3425	0.0417	0.0144
6	D_hansenii	C_guilliermondii	0.6842	3538	0.0221	0.1883
7	D_hansenii	L_elongisporus	0.6628	3345	0.0083	0.6275
8	D_hansenii	C_parapsilosis	0.6561	3324	0.0257	0.1378
9	D_hansenii	C_tropicalis	0.6104	3358	0.0073	0.6685
10	E_gossypii	S_pombe	0.7616	1536	0.0837	0.001
11	E_gossypii	Y_lipolytica	0.7114	2441	0.0297	0.1414
12	Y_lipolytica	M_grisea	0.6751	2464	0.041	0.0418
13	Y_lipolytica	A_nidulans	0.7072	2515	0.0162	0.4156
14	Y_lipolytica	C_immitis	0.7109	2584	0.0021	0.9117
15	Y_lipolytica	S_pombe	0.7271	1270	0.0404	0.1495
16	S_pombe	S_japonicus	0.6551	1461	0.0549	0.0358
*17	***C_dubliniensis***	***C_albicans***	***0.2893***	***3796***	***0.2176***	***6.30E-42***
*18	***C_dubliniensis***	***C_tropicalis***	***0.5522***	***3514***	***0.1029***	***9.80E-10***
*19*	*C_tropicalis*	*C_parapsilosis*	*0.6059*	*3390*	*0.0426*	*0.013*
20	C_tropicalis	L_elongisporus	0.6257	3442	-0.0227	0.1818
*21	*N_crassa*	*C_globosum*	*0.5842*	*2369*	*-0.0085*	*0.6757*
*22	*U_reesii*	*C_immitis*	*0.5293*	*917*	*-0.0513*	*0.1201*
*23	*A_nidulans*	*A_terreus*	*0.6364*	*1121*	*0.037*	*0.2147*
24	H_capsulatum	C_immitis	0.6839	847	0.0356	0.2999
*25	*H_capsulatum*	*U_reesii*	*0.6655*	*393*	*0.0034*	*0.9459*
*26	*L_elongisporus*	*C_parapsilosis*	*0.6363*	*3152*	*0.0265*	*0.1366*

The only species pairs with a significant Pearson correlation (<0.001) are those among *C. dubliniensis, C. albicans*, and *C. tropicalis *(shown in bold). This indicates that virtually all fungal genomes have uniform mutation rates. Average divergence is the fraction of all aligned 4-fold sites which differ between the two species. Species pairs in italics and marked with a * have at least 10% fewer substitutions than would be expected at mutational saturation.

**Figure 3:**
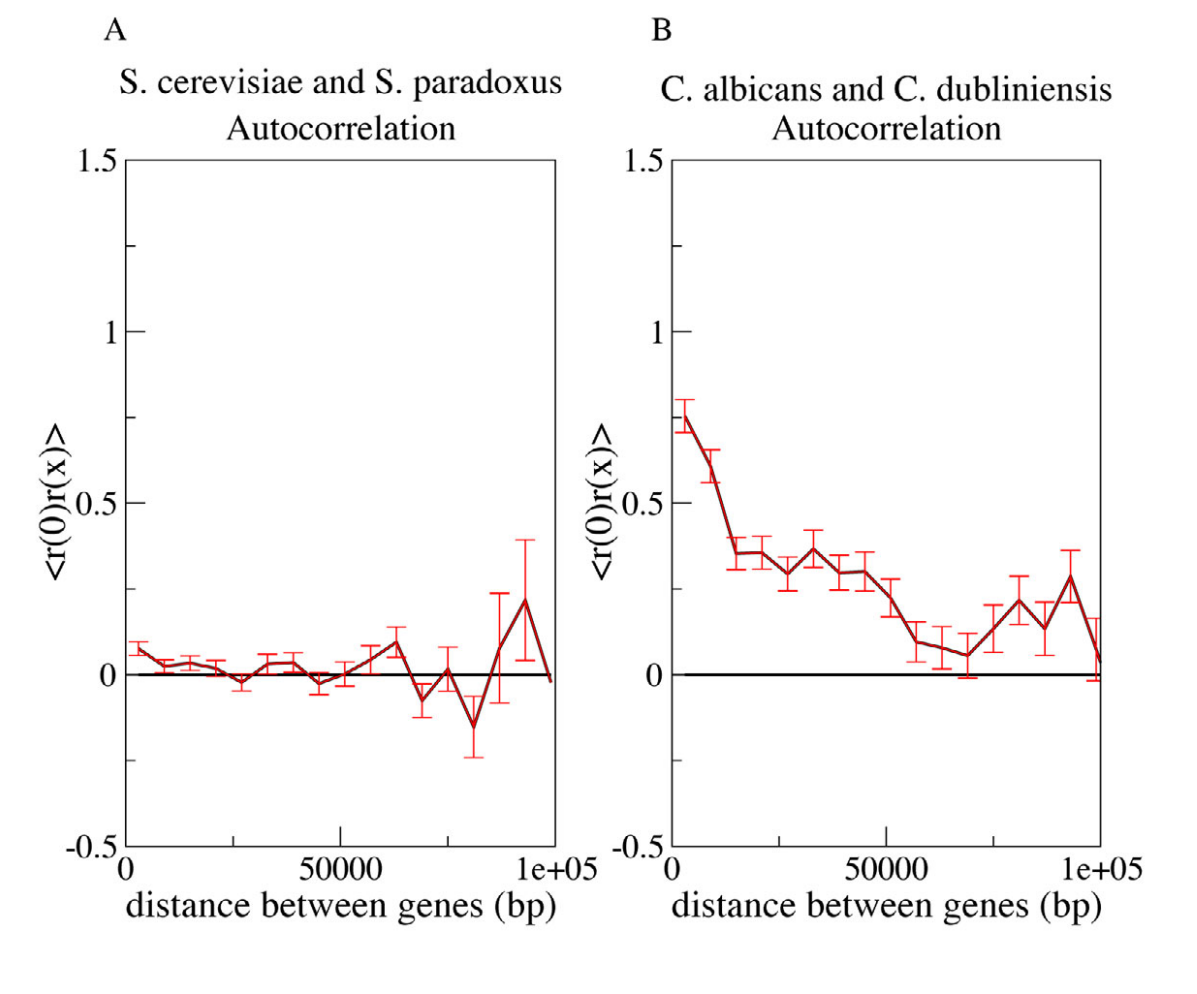
**Autocorrelation of normalized substitution rates *r *for the *S. cerevisiae/S. paradoxus *(a) and *C. dubliniensis/C. albicans *(b) pairwise analyses**. The *C. dubliniensis/C. albicans *plot shows that substitution rates of neighboring genes are correlated up to 50 kb apart. This contrasts with the lack of correlation at any distance for the *S. cerevisiae/S. paradoxus *plot. Analogous graphs were produced for all fungal species pairs, and no significant autocorrelation was found, except for the *C. dubliniensis/C. albicans *and *C. tropicalis/C. dubliniensis *comparisons.

More generally, we computed an autocorrelation function *<r(x)r(0)>*, where *r(0) *is the normalized substitution rate of a gene and *r(x) *is the normalized substitution rate of a gene that is *x *base pairs downstream of the first gene [9]. Because the rates have been constructed to be centered around *r = 0*, we would expect *<r(0)r(x)> ~ 0 *if there are no regional rate biases (see Methods). We detected no apparent autocorrelation for species pairs from any of these 22 genomes (Data for the *S. cerevisiae-S. paradoxus *species pair, which is typical, is shown in Figure 3a). Thus these fungal species do not exhibit the regional mutation structure that has been observed in mammals.

### Three Candida yeasts have heterogeneous mutation rates

Species pairs from two fungal clades [(*C. albicans, C. dubliniensis, C. tropicalis*) and *(N. crassa, C. globosum*)] were found to have *r *distributions that do not fit the Normal curve. These species have wider *r *distributions (2.07 ≤ σ ≤ 2.20) (Figures 1c and 2).

For the three *Candida *species, autocorrelation analysis indicates that substitution rates are significantly correlated for genes within 50,000 base pairs of each other (Figure 3b). This implies that *C. albicans *regional biases extend over scales encompassing ~20 genes, as the spacing between genes is 2300 bp [17]. The neighboring gene Pearson correlations were also found to be highly significant (*C. dubliniensis/C. albicans *Pearson correlation = 0.218, p = 6.4 × 10^-42^; *C. tropicalis/C. dubliniensis *Pearson correlation = 0.103, p = 9.8 × 10^-10^). These regional biases are not due to a CpG dinucleotide effect. When we excluded CpG sites from the analysis, neighboring genes were still observed to have correlated substitution rates (Pearson-correlation = 0.1096, p < 10^-8^). These *Candida *species translate CUG as serine instead of the usual leucine, and it might be hypothesized that this alternate codon usage is relevant to the rate inferences [18]. However, ignoring such codons does not significantly diminish the correlation (*C. dubliniensis/C. albicans *Pearson correlation = 0.208, p = 10^-27^).

### *N. crassa *and *C. globosum *substitution rates are skewed by strong codon usage bias

As in the Candida species, substitution rates between *N. crassa *and *C. globosum *give an *r *distribution wider than the Normal Gaussian. However, there is no significant correlation (Pearson correlation = -0.0085, p = 0.6757) between neighboring genes, and the autocorrelation plot is similar to those of the fungi with uniform rates (data not shown). These results suggest that the wide rate distribution (σ = 2.07) is due to pressures on individual genes, rather than regional effects.

We hypothesized that the large σ without apparent regional correlation could be due to increased selection on the silent sites of *N. crassa *and *C. globosum *genes. Such selection could cause some genes to have more extreme conservation and thus broaden the rate distribution. To test this, we considered the codon usage bias in these species, as this is the best understood type of silent site selection in yeasts.

**Figure 4:**
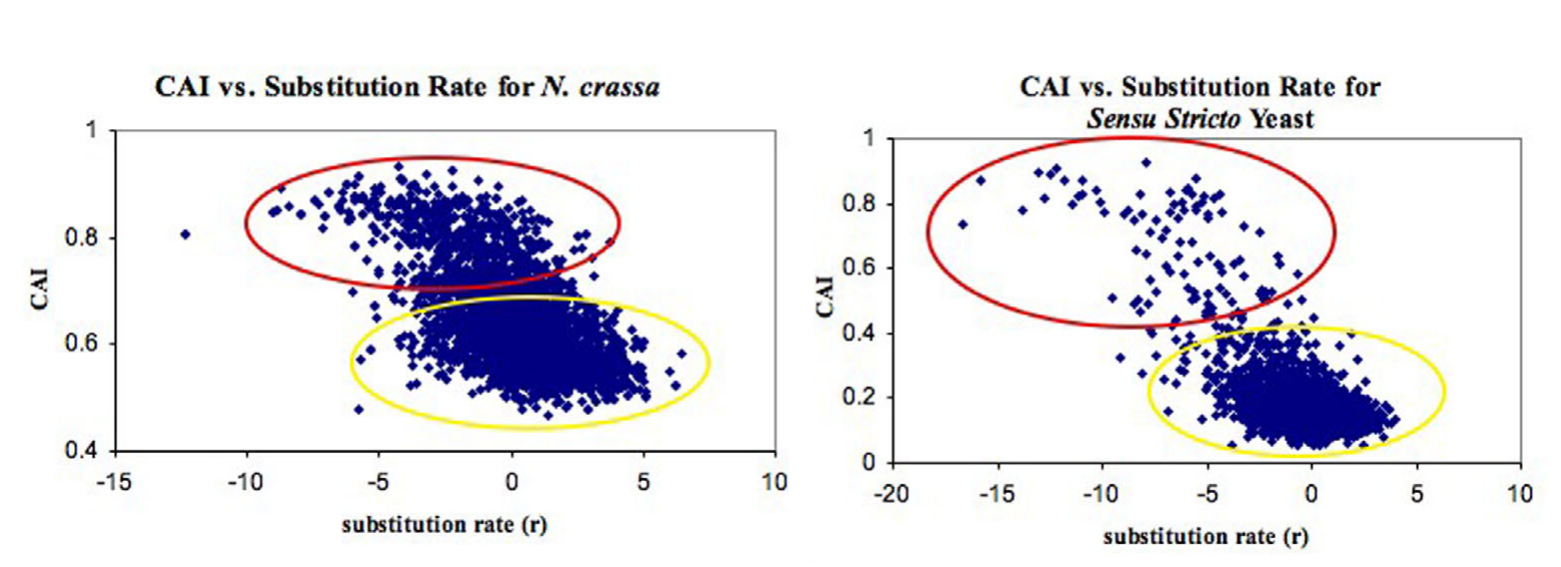
**Graphs of Codon Adaptation Index (CAI) vs. normalized substitution rates**. Data for the *sensu stricto *yeasts (*S. cerevisiae, S. bayanus, S. mikatae, S. paradoxus*) (right) and for *N. crassa-C. globosum *(left) are both shown. Although the *r *distribution for *N. crassa-C. globosum *(Fig 1c) is wider than a Normal distribution, this appears to be due to a large number of genes under codon usage selection, rather than regional mutational biases. Genes under codon usage bias tend to have low z-scores and high CAI values (red ellipse), while the remaining genes have low CAI values and *r *values centered around zero (yellow ellipse). *N. crassa *has a much larger number of genes in the cluster likely to be under codon usage selection (949 genes with CAI≥0.70, red ellipse) than does *S. cerevisiae *(121 genes with CAI≥0.40), suggesting that more genes are under codon usage selection in *N. crassa*.

Our results indicate that codon usage selection is a more widespread effect in *N. crassa *than in the *sensu stricto *yeasts (Figure 4) (or than in *C. albicans*, Additional file 2). Codon adaptation index (CAI) [19] values were calculated for *N. crassa *and *S. cerevisiae *based on their respective codon biases. *N. crassa *genes generally have higher CAI values (median 0.63) than *S. cerevisiae *genes (median 0.14). In each of the graphs, there is one main cluster of genes with *r *scattered around zero and with low CAI values. There is also another group having negative *r *and high CAI, indicating codon usage selection. *N. crassa *has more genes in the group apparently subject to codon usage selection (949 genes above CAI = 0.7) than *S. cerevisiae *(121 genes above CAI = 0.4). This suggests that codon usage selection affects more genes in *N. crassa/C. globosum*, which could explain the larger σ for these genomes (See Discussion).

### All mammals have heterogeneous mutation rates

**Figure 5:**
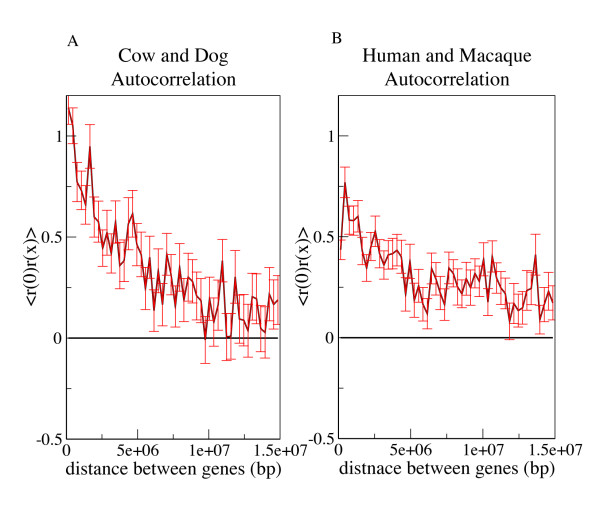
**Autocorrelation of normalized substitution rates *r *for cow-dog (a) and human-macaque (b)**. In each species pair, nearby genes have correlated silent site substitution rates even at distances up to 10 Mb. These lengthy regional biases are typical of mammalian genomes, and are much larger than those of the *Candida *fungi.

**Figure 6:**
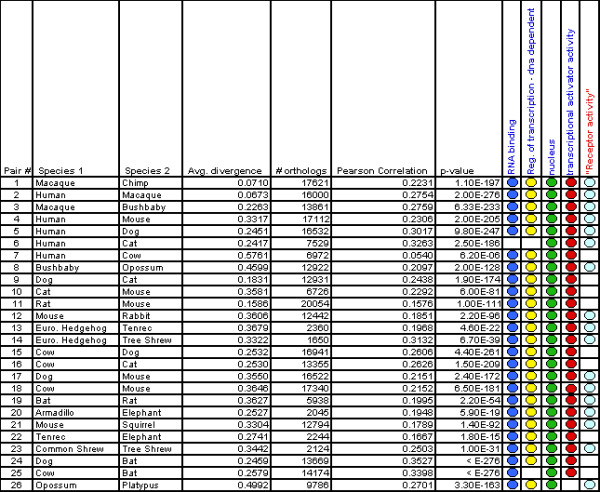
**Pearson correlation of substitution rates between neighboring mammalian genes and evidence of selective pressures**. 20 mammalian species and 26 species pairs are shown. In striking contrast to the fungi, every mammalian comparison gives a highly significant correlation between rates of neighboring genes (column 7). (Right) Similar types of genes have unusually low or unusually high silent substitution rates across the mammals, suggesting universal selection pressures. Genes in the categories RNA binding, regulation of transcription – DNA dependent, nucleus, or transcriptional activator activity (blue text) have unusually low silent substitution rates in virtually every mammalian species pair, as marked by ovals. Gene associated with receptor activity have unusually high silent substitution rates (red text) in most mammalian species pairs.

All 20 mammalian species were found to have strong regional mutation rates, as evidenced by the wide mammalian rate distributions (1.80 ≤ σ ≤ 2.23) (Figures 1a and 2). The Pearson correlation (Figure 6) of neighboring genes was found to be significant for every pairwise mammalian comparison. Autocorrelation analysis indicates that the length scales of mutational blocks are similar in the various branches of the mammalian phylogeny as well. For example, in the cow/human comparison (Figure 5), the autocorrelation graph suggests mutational blocks along each chromosome as large as 10 Mb, similar to the length scale that has been observed in mouse and human.

The regional variations are also apparent from correlation analysis of ancestral repeats, another largely neutral dataset scattered throughout the genome [20]. We analyzed 16-way vertebrate multispecies alignments from genome.ucsc.edu to obtain a set of ancestral repeats aligned orthologously across the species pairs (human, macaque), (mouse, rat), (elephant, tenrec), and (dog, cow). For each species pair we observed that an ancestral repeat's substitution rate is correlated with the neighboring ancestral repeat along the genome (Table 2). In each case the significance of the correlation was at the limit of computational precision (p < 10^-276^). The rate distributions for repeats are also wider than the Normal, with standard deviations ranging from 1.41 (elephant-tenrec) to 1.96 (human-macaque) (Figures 1a and 1c).

**Table 2:** Correlation of substitution rates in neighboring ancient repeats.

species1	species2	# of repeats	Pearson Correlation	p-value
Human	Macaque	5081426	0.32192	< 10^-276^
Dog	Cow	1378699	0.18829	< 10^-276^
Elephant	Tenrec	248577	0.12158	< 10^-276^
Rat	Mouse	231283	0.1292	< 10^-276^

In all four mammalian pairs considered (human-macaque), (dog-cow), (elephant-tenrec), and (rat-mouse), the correlations were extremely significant (<10^-276^). This supports the widespread heterogeneity of mutation rates in mammalian genomes.

### Insects have heterogeneous mutation rates

The fungal species we have studied are at phylogenetic distances generally larger than those for the mammalian species. As species approach saturated divergence, mutation rate inferences become more uncertain. Therefore one might be concerned that the greater divergences for fungal species pairs could be obscuring regional effects. This hypothesis can be tested using insect genomes. *Drosophila melanogaster *and *Drosophila pseudoobscura *are about as diverged (4-fold divergence = 0.514) as most fungal pairs, but are more diverged than the mammalian species pairs. This is also true for the mosquitoes *Aedes aegypti *and *Anopheles gambiae *(4-fold divergence = 0.632).

However, unlike most fungi, flies and mosquitoes show clear regional effects. *D. melanogaster *and *D. pseudoobscura *have significant rate correlations between neighboring genes (Pearson correlation = 0.1642, p = 10^-58^) and have a wide *r *distribution (σ = 2.152). Autocorrelation analysis indicates that correlations persist out to at least 1 Mb (data not shown). This conclusion of mutational heterogeneity is consistent with studies of transposon mutation within *D. melanogaster *[21]. Rate correlations between neighboring genes are also apparent in comparisons of *Aedes aegypti *and *Anopheles gambiae *(Pearson correlation = 0.2099, p = 10^-89^, σ = 1.804). These insect correlations are stronger than that for *C. albicans-C. dubliniensis*, despite the fact that the two *Candida *species have a lower divergence (0.289). We conclude that the general lack of correlations for the fungi is unrelated to the magnitude of their phylogenetic separations.

### Genes with low substitution rates throughout the mammalian phylogeny

While these themes of mutational heterogeneity and uniformity can describe much of the behavior of silent sites in genes, our data also reveal certain types of genes whose silent substitution rates suggest selective pressures. For example, it has been observed that genes involved in gene regulation tend to have few silent substitutions among human, mouse, cow, and dog, while genes interacting with the extracellular environment tend to have many substitutions [2,22]. We have tested the generality of such observations throughout the mammalian phylogeny.

For each mammalian species pair, a list of Gene Ontology [23] categories with unusually high or low silent site substitution rates was calculated. Unusual categories were identified by aggregating the *r *values from all genes in the GO category into a z-score *z*_*GO *_(see Methods). Positive *z*_*GO *_values indicate that a category has above average substitution and negative values indicate below average substitution.

We observed four GO categories with unusually low substitution rates (*z*_*GO *_negative and statistically significant) across almost all mammalian species pairs (Figure 6). For the categories "regulation of transcription, DNA-dependent," "RNA binding," and "nucleus", every mammalian species is involved in at least one pair in which these categories have a significantly depressed rate. The category "transcriptional activator activity" has a low substitution rate in every comparison except for the outlier species opossum-platypus. As an illustration of the strength of this effect, the GO category "regulation of transcription, DNA-dependent" contains 1224 genes with a *z*_*GO *_of -8.893 (p = 10^-18^) for the mouse-human species pair, and for the cow-dog species pair the category has a *z*_*GO *_of -7.182 (p = 10^-12^). It cannot be ruled out that RNA binding and DNA binding genes sit in slowly mutating regions in every species. However, given the large number of diverse lineages involved, as well as previous studies indicating that some of these genes contain ultraconserved blocks more than a hundred bp long [24], a simpler explanation is that these genes contain functional silent sites (see Discussion). Interestingly, even in the *sensu stricto *yeasts (*S. cerevisiae-S. paradoxus*) and (*S. cerevisiae-S. bayanus*), the categories RNA binding and nucleotide binding have unusually low substitution rates. Thus silent sites in nucleotide binding genes are likely to be under selection in both unicellular and multicellular eukaryotes. This does not seem to merely be due to these yeast categories containing genes under codon usage selection. In the comparison between *S. cerevisiae *and *S. bayanus*, the overall *z*_*GO *_for the nucleotide binding category is -7.320, and it is -6.686 (still highly significant) when all genes in the glycolysis GO category are removed.

### Genes with high substitution rates throughout the mammalian phylogeny

It has previously been observed that genes which interact directly with the environment have high silent substitution rates in mouse-human comparisons, and we found that this is generally true among the mammals. While we found no GO category with unusually high substitution rate (z_GO _positive and statistically significant) in every mammalian species pair, there was considerable similarity across species. 17 out of the 26 mammalian species pairs have at least one high z_GO _category with "receptor activity" in the description (Figure 6). 15 have "olfactory receptor activity", 23 have "metabolic process", 10 have "membrane", and 9 have "sensory perception" and/or "sensory perception of smell". These annotations repeatedly occur in distinct clades. For example, the category "olfactory receptor activity" has a significant z_GO _for the species pairs armadillo-elephant, mouse-rabbit, opossum-platypus, human-macaque, and common shrew-tree shrew, among others. Such genes appear to often be clustered in tandem arrays in primate genomes, though such clustering is not as prevalent in other mammals.

The primate clade has strong consistency in the categories with high *z*_*GO*_. We found 10 high *z*_*GO *_categories in common among the four primate species pairs (macaque-bushbaby), (human-bushbaby), (human-macaque), and (macaque-chimp). However, we found no such categories consistent throughout species pairs of the clade (rat, mouse, squirrel, rabbit) or the clade (cat, dog, cow, bat, hedgehog, shrew). This may be related to the fact that primates are a more closely related group.

Could the high substitution rates in receptor genes be related to diversifying selection on the protein sequences of such genes? This could be responsible if protein changes have reduced the cross-species alignability of such genes and artifactually increased silent site substitution rates in misaligned regions. To test this, we recalculated rates using only 4-fold sites at least 3 codons away from any indel in the alignment. With these rates, we still observed 16 mammalian lineages having at least one "receptor activity" category with an unusually high z_GO_. This robustness shows that the high substitution rates in receptor genes are not due to diversifying selection at the amino acid level but are instead a separate effect.

### Genes with unusual substitution rates in the fungi

As discussed above, codon usage selection is the clearest determinant of low silent site substitution in the fungi. The GO category "translation" has the lowest z_GO _in the *sensu stricto *yeasts (*S. cerevisiae-S. bayanus *z_GO _= -11.995), and it also has a low z_*GO *_in *C. albicans-C. dubliniensis *(z_GO _= -4.617). Six low-rate categories are consistent among (*C. albicans-C. dubliniensis*), (*C. dubliniensis-C. tropicalis*), (*S. cerevisiae-S. bayanus*) and (*S. cerevisiae-S. paradoxus*): "translation", "unfolded protein binding", "structural constituent of ribosome", "ribosome", "glycolysis", and "chitin- and beta-glucan-containing cell wall"), and these categories are largely consistent with codon usage selection.

However, we also found several other *C. albicans-C. dubliniensis *categories with much stronger silent site conservation than "translation," suggesting other types of silent site selection. The categories with the lowest z_GO _are GO:0005625 soluble fraction (*C. albicans-C. dubliniensis *z_GO _= -12.468) and GO:0030445 yeast-form cell wall (*C. albicans-C. dubliniensis *z_GO _= -8.81). Other categories with low substitution rates (GO:0044416 induction by symbiont of host defense response, z = -7.876; GO:0051701 interaction with host, z_GO _= -8.547) suggest that *Candida *genes involved in virulence have functional silent sites. It is unlikely that these categories have silent site conservation only because of codon usage selection. After removing all genes also found in categories involving glycolysis from the GO groups "soluble fraction", "yeast-form cell wall" and "interaction with host", each category maintains a significant negative z-score.

Few fungal gene categories were found to have high z_GO _values. This is not surprising for the *sensu stricto *yeasts, given their mutational uniformity. We found only one category with a significant positive z-score in both *S. cerevisiae-S. bayanus *and *S. cerevisiae-S. paradoxus*, and its p-value is marginal (transcription factor activity, Bonferroni-corrected p-value = 0.9216). We also found few *Candida *categories with unusually high substitution rates, despite their mutational heterogeneity. *C. albicans-C. dubliniensis *and *C. dubliniensis-C. tropicalis *share one category with a significant positive z_GO _("response to pH"), and *C. albicans-C. dubliniensis *has the additional category "cell adhesion". Interestingly, the "cell adhesion" category has a higher average nonsynonymous substitution rate (K_a_) than the genome-wide average (0.2234 vs. 0.0857). Thus these cell adhesion genes are analogous to mammalian receptor genes, which tend to have both high synonymous and non-synonymous substitution rates [2].

## Discussion

Based on our examination of 20 mammalian species, we conclude that all mammals have regional biases of neutral mutation rates. While the factors controlling these regional biases are still not well understood [1], our findings indicate that any causal variables must prevail throughout the mammalian phylogeny. In contrast, we find that almost all fungi have a uniform neutral mutation rate. It is worth noting that our fungal phylogeny covers a broader range of divergence than the mammals, giving this shared behavior more significance. However, the monophyletic group of *Candida *species (*C. albicans, C. dubliniensis*, and *C. tropicalis*) is an exception to the other fungi. Rate distributions and gene-to-gene correlations indicate that these species have heterogeneous mutation rates along their genomes, a trait which has not previously been observed outside species with much larger genomes. Previous *C. albicans *SNP studies have indicated hotspots of polymorphism, supporting our conclusion of regional biases [25,26].

What characteristic sets these three yeasts apart from the others? Of relevance is a recent study showing that *S. cerevisiae *polymorphisms arise more often in regions that replicate late in the cell cycle (G. Lang and A. Murray, personal communication). This finding can be reconciled with our observation of uniformity of *Saccharomyces *divergence rates, if replication timing is mutable – i.e. provided the replication timing of a locus switches faster than a polymorphism fixes in the population. However, in species where the replication timing is static, mutational heterogeneities would manifest as heterogeneities in the cross-species substitution rates. An experiment that compares switching of replication timing in *Candida *and *Saccharomyces *loci would be an exciting way to test this prediction.

A comparison of *N. crassa *and *C. globosum *also gives an excessively wide distribution of normalized substitution rates, but this is due to stronger codon usage selection, rather than regional mutation effects. This strong codon usage selection is more apparent in the context of the mutational defense mechanism RIP [27] that *N. crassa *employs against selfish DNA. RIP (repeat-induced point mutation), inactivates selfish DNA by inducing G:C to A:T mutations in sequences that occur repetitively in the genome. This process affects all duplicated regions – except for ribosomal RNA genes, even though these occur in large copy number [28]. This observation is consistent with ribosomal genes being under strong codon usage selection that shields them from the effects of RIP. Meanwhile, RIP drives other duplicated genes in *N. crassa *to unusually high substitution rates, which would increase the genes with high *r *in the rate distribution.

We have observed that genes interacting with the extracellular environment, especially receptors and olfactory genes, tend to have high substitution rates in many mammalian lineages. This is in agreement with previous findings in comparisons of human and mouse [2], and it suggests a recurring interplay between local mutation rate and receptor genes. Residence in a region of high mutation rate may be useful for speeding up the diversification of receptor protein sequences and improving the evolvability of the organism [29]. An alternative explanation is that the silent sites could themselves be under diversifying selection, though it is unclear why receptor gene silent sites would be more disposed to such an effect.

We have also found that the silent sites of genes associated with RNA binding and DNA binding are strongly conserved in not only every mammalian species but also the *sensu stricto *yeasts. This expands similar findings in which mammalian genes have been found to have blocks over a hundred bp long strongly conserved across the mouse, rat, human, and dog genomes [22,24,30]. Given the large evolutionary divergences spanned by these species and the tendency of mutation rates to change over time (H. Imamura, J. Karro, and J. Chuang, in preparation), it seems unlikely that these consistently low substitution rates are due to regional background effects in every species. A simpler explanation is that synonymous sites are commonly functional in such genes throughout the eukaryotic phylogeny.

## Conclusion

Among the 51 species studied in this work, all characterized mammals and insects exhibit regional biases in the mutation rates along their genomes, based on the substitution rates in gene synonymous sites. In contrast, virtually all fungi show uniform mutation rates. One fungal clade, the *Candida*, is the exception, as the only fungal clade with regional mutational biases. Further analysis of these genomes should be valuable for revealing what features control local mutation rates. Also, in decomposing neutral and selective effects at synonymous site we found classes of genes whose synonymous sites have been commonly influenced by selection. Most notably, there appears to have been widespread purifying selection in regulatory genes and frequent diversifying selection in receptor genes throughout mammalian history.

## Methods

### Ortholog generation

For the fungal analysis, FASTA files of coding regions and an ortholog tree listing predicted gene relationships between all fungal species were obtained as described in [31]. Mammalian and mosquito genes were obtained using the ENSEMBL BioMart (release 45) database. Fly CDS and peptide files were downloaded from FlyBase (version FB2006_01). For the fly species, the amino acid sequences were run through BLAST and tagged as true orthologs if they were each other's mutual best hit when applying BLASTALL with a worst-case E-value cutoff of 10^-10^.

### Calculation of substitution rates

Our substitution rate calculations parallel those in previous works [2,9]. Nucleotide sequences of orthologous coding regions were translated into amino acid residues, aligned using CLUSTALW, and then back-translated to determine the aligned DNA sequence. The fourfold synonymous sites – the third base in a codon for which the amino acid is determined by the first two positions – were analyzed. If a sequence contained fewer than 20 fourfold sites before occurrence of a stop codon, the entire sequence was discarded. To ensure equivalent sequence context, only fourfold sites for which both the preceding and succeeding base matched for the two species were considered.

The raw neutral substitution rate was calculated based on the fraction of observed differences at silent sites within a gene. Individual gene rates were then normalized in order to correct for the finite-size of each gene, and this new rate was defined to be

r = (p-<p>)/s(N),

where p is the observed 4-fold substitution rate for the gene in question and <p> is the average substitution rate for all ortholog pairs for the two species in question. s(N) is the expected standard deviation for a gene with N independent fourfold sites, i.e. s(N) = (<p>(1-<p>)/N)^1/2^. The distribution of normalized substitution rates would be expected to follow a normal Gaussian (f(x) = e^-x*x/2^/√(2π)) if each fourfold site was mutating at the same rate and independently of each other. This z-score normalization corrects for the finite-size effects that result from genes having different numbers of 4-fold sites.

One caveat to our approach (and any measurement of sequence divergence) is that divergence measurements lose power when comparing species with saturated divergence. However the large majority of species pairs described here are at sequence divergences considerably below saturation.

For every species pair described, we calculated the expected divergence at saturation as $1-\sum_{i=ACGT}f_{i}^{\left(1\right)}f_{i}^{\left(2\right)}$, where $f_{i}^{\left(1\right)}$ and $f_{i}^{\left(2\right)}$ are the frequencies of base *i *in species 1 and 2, respectively. The variation in this quantity is relatively small, ranging from 0.6634 (*C. dubliniensis-C. tropicalis*) to 0.7519 (opossum-platypus). As is visible from Tables 1 and 2, all of the mammalian comparisons are considerably below saturation. Each of the mammalian comparisons is at least 600 standard deviations less substituted than what would be expected if every 4-fold site were substituted independently at the saturated probability. While the fungi are generally at greater distances, a number are still below the saturated expectation. All yeast comparisons (with the exception of *E. gossypii-S. pombe*) are several dozen standard deviations below the saturated rate expected from an independent-sites model. More strongly, there are 10 species pairs with divergences at least 10% below the saturated substitution level (Table 1). These include representatives from several independent clades all with substitution rates below 0.60, including *U. reesii/C. immitis*, *N. crassa/C. globosum*, the *Saccharomyces *yeasts, and the *Candida *yeasts.

A potential concern is that sequence alignments could be less reliable for the fungal species, given their large sequence divergences. We therefore recalculated all of the fungal substitution rates using only high quality regions of the pairwise alignments, which we defined to be a concatenation of blocks containing a minimum of 10 consecutive aligned amino acids with no gaps. We then compared the original substitution rates to those rates obtained from the restricted high quality alignments. For the 26 pairs, all had a correlation of greater than 0.80 (p-values < 10^-146^) with the original results, and 16 had correlations greater than .90. Thus we do not expect that alignment quality is a concern.

### Correlation calculations

We tested whether or not neighboring genes had similar substitution rates by calculating a Pearson correlation between the rate of gene *r(0) *and the rate of gene *r(x) *which is located *x *base pairs downstream. Gene pairs in orthologous blocks up to the 35^th ^gene downstream from the starting gene were considered. Blocks were determined by genes located on the same chromosome (scaffolds were used when chromosomal data was not available). Correlations were measured twice, in each case using location data from one of the species, except in cases where location data was available in only one species. For the fungi, the data for each pairwise calculation was binned into 50 uniformly spaced groups covering *x *= [0, 300000] and averaged over each bin to determine the autocorrelation function *<r(0)r(x)>*. Error bars were assigned based on the standard deviation of the values in each bin. For the larger genomes of mammals and insects, data was binned into 200 groups where *x *ranged from 0 to 15 Mb.

### CAI

CodonW (Peden 1999) was downloaded and used to calculate the CAI values for the fungal species. The input file for each species was a CDS FASTA file of all genes (predicted and known) and the background CAI was set to *Saccharomyces cerevisiae *or the *sensu stricto *calculation in Figure 4. The *S. cerevisiae *sequence and codon preferences were used to compute the CAI values. The EMBOSS package was also downloaded locally. This includes codon usage tables for a number of species including *N. crassa *[32] and *C. albicans*. This table was used to calculate the CAI for the genes in Figure 4 and Additional file 2. *Sensu stricto *substitution rates in Figure 4 were taken from [9].

### z-score calculation for GO analysis

GO assignments were taken from ENSEMBL annotations of the orthologous human gene. While this eliminates orthologs that do not have an ortholog in human, only a small minority of genes are affected by this problem. For example, of 7,959 GO annotated gene products for cow, only 447 (1.5%) do not have a human ortholog. A z-score and p-value was assigned to each GO category based on the substitution rates for all of the genes included in the category.

The z-score (z_GO_), calculated for each GO category based on the substitution rates of all members within the group, was defined to be

z_GO _: = (<r>_GO _- <r>_all_)/(s_all_/√N_GO_)

where <r>_GO _is the average substitution rate for the genes in the GO category and <r>_all _is the average substitution rate for all genes. s_all _is the standard deviation of the <r>_all _data set and N_GO _is the number of genes in that particular GO category. The p-value for the z_GO _calculated for an ontology group is the probability that a Gaussian-distributed variable would be represented by a value ≥ z_GO_. The p-value cutoff was set to be 1/X where X is the number of GO categories (with a minimum of 5 genes). The significance was then expressed in terms of -log_10_(p-value).

## Authors' contributions

AKF performed sequence and data analysis and drafted the manuscript. BBT obtained and analyzed the fungal data and contributed to the writing of the manuscript. JHC conceived of the study, contributed to the data analysis, and contributed to the writing of the manuscript. All authors read and approved the final manuscript.

## Supplementary Material

Additional file 1 Phylogeny containing the yeast species. Click here for file

Additional file 2 Comparison of codon usage effects in *N. crassa *and *C. albicans*. Click here for file
