# Supplementary figures and images for: Measuring the prevalence of regional mutation rates: an analysis of silent substitutions in mammals, fungi, and insects
Source: BMC Evol Biol. 2008 Jun 27;8:186. doi: 10.1186/1471-2148-8-186 (PMC2447844; doi:10.1186/1471-2148-8-186)

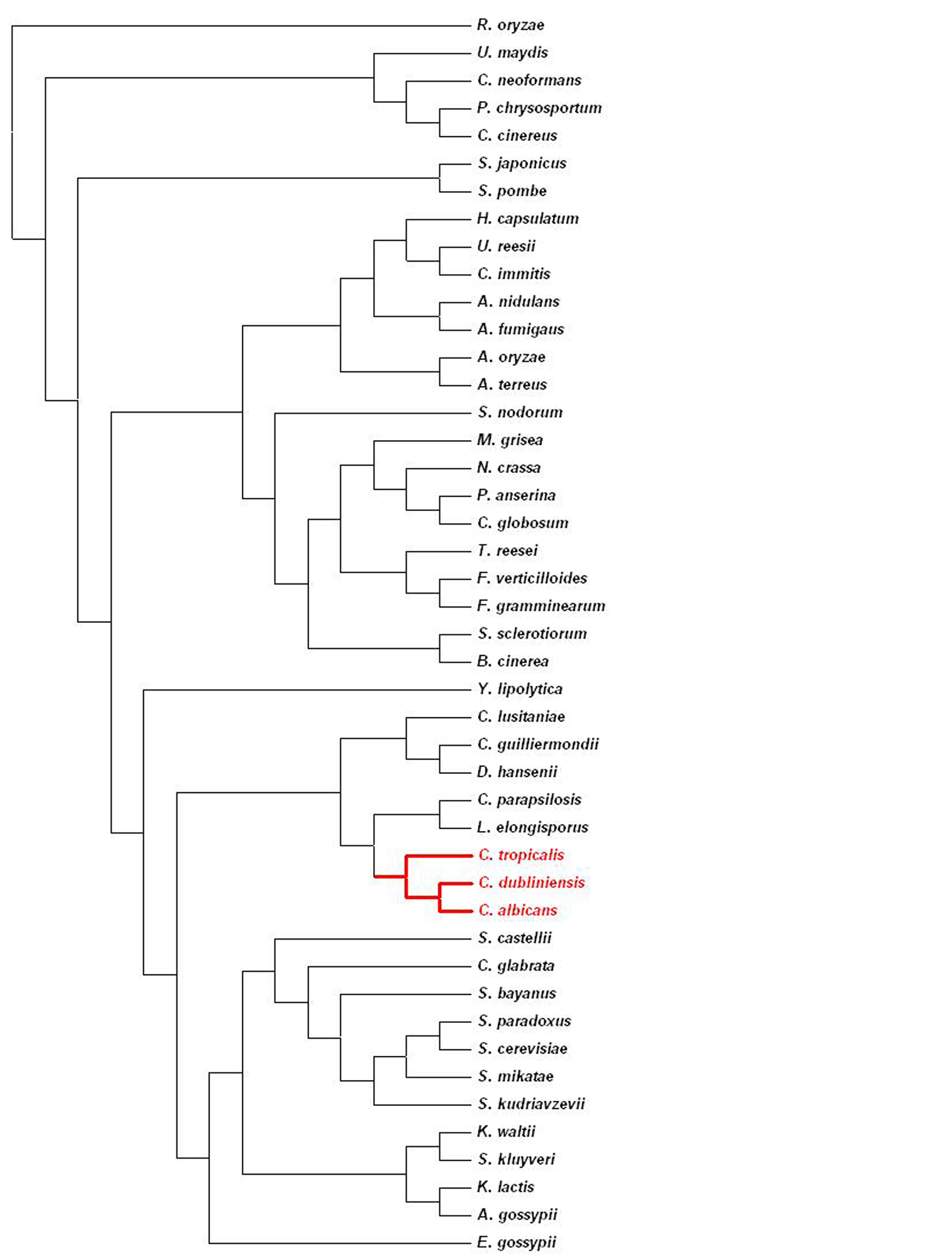

Supplement: Additional file 1 — Phylogeny containing the yeast species. [file 1471-2148-8-186-S1.tiff]

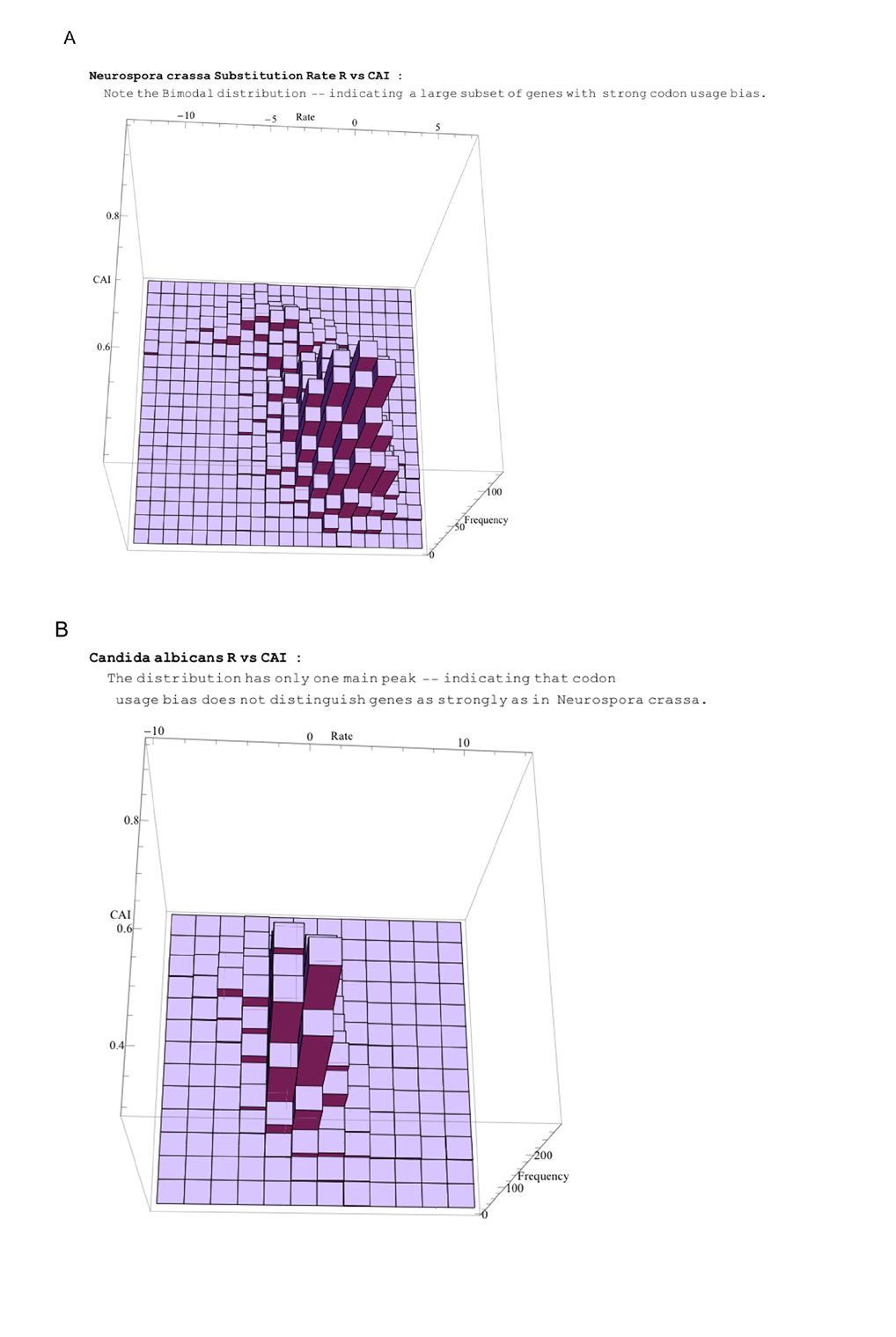

Supplement: Additional file 2 — Comparison of codon usage effects in N. crassa and C. albicans. [file 1471-2148-8-186-S2.tiff]
